# Development and Implementation of an Internal Quality Control and External Quality Assessment Information System for a Regional Medical Laboratory Center: Pilot Design and Implementation Study

**DOI:** 10.2196/77043

**Published:** 2025-12-09

**Authors:** Dayang Chen, Hongmei Mo, Yingying Zhang, Junlong Qin, Lijun Zhang, Changxian Jiang, Fuming Li, Xiuming Zhang, Tong Ou

**Affiliations:** 1 Department of Medical Laboratory The Third Affiliated Hospital of Shenzhen University Shenzhen China; 2 Department of Medical Laboratory Center Shenzhen Luohu Hospital Group Shenzhen China; 3 Department of Medical College Shantou University Shantou China; 4 Bio-Rad Laboratories (Shanghai) Co, Ltd Shanghai China; 5 Department of Pathology The Hong Kong University - Shenzhen Hospital Shenzhen China

**Keywords:** internal quality control, external quality assessment, quality management, laboratory information system, regional medical laboratory center

## Abstract

**Background:**

The Regional Medical Laboratory Center (RMLC) integrates laboratory departments from hospitals with different levels to optimize resource allocation, enhance testing efficiency, and promote the sharing of professional expertise. Despite these benefits, ensuring consistent quality control (QC) across the RMLC presents a significant challenge due to diverse equipment, procedures, and staff expertise levels. To address these challenges, this study provided an information tool called iLab, developed by Shenzhen Huikang Information Technology Co, Ltd, for managing internal quality control (IQC) and external quality assessment (EQA).

**Objective:**

This study aimed to develop and implement an integrated informatics platform managing IQC and EQA across RMLC.

**Methods:**

We integrated 4 software programs to manage IQC and EQA, laboratory information system (LIS) supplied by Shanghai Tengcheng Medical Tech-Info Co, Ltd; Quality Control Box (QCBOX) and Quality Control EQA Center (QCEC) provided by Bio-Rad Laboratories (Shanghai) Co, Ltd; and the iLab electronic recording system. The platform centralizes QC data processing, with QCBOX and QCEC handling operational workflows, while iLab oversees documentation, analytics, and approval processes. It was successfully deployed in August 2023 across a pilot network comprising 5 district hospitals, 1 central laboratory, and 4 community health stations in Shenzhen's Luohu District.

**Results:**

By leveraging the LIS-QCBOX data flow, the platform streamlined the entire IQC process, which incorporated interlaboratory comparisons and iLab-facilitated postanalysis management. Concurrently, EQA was enhanced through electronic documentation, multirule analysis of acceptable data, regional proficiency testing via QCEC, and measurement uncertainty (MU) estimation. By September 2025, the platform supported 114 users, integrating 133 instruments and 859 active QC items, and generated 2331 monthly IQC analysis reports, 340 EQA multirule analyses, and 289 MU reports. Comparative analysis revealed marked improvements in key metrics from 2023 (preimplementation) to 2024 (postimplementation): the IQC implementation rate rose from 97.79% to 99.87% (P<.001), the report error rate decreased from 0.048% to 0.027% (P<.001), and the intralaboratory turnaround time compliance rate increased from 95.49% to 95.71% (P<.001). The annual EQA unacceptable rate dropped from 0.34% in 2023 to 0% in 2024. The number of ISO (International Organization for Standardization) 15189–accredited test items increased from 203 in 2022 to 206 in 2024.

**Conclusions:**

The iLab system establishes a scalable framework for standardized IQC and EQA management in RMLC, demonstrating significant potential to enhance regional laboratory quality.

## Introduction

The Luohu Hospital Group, comprising 5 district hospitals and 43 community health stations, serves as a model for integrated urban health care systems in China. China’s National Health Commission endorsed the Luohu model as the template for building a people-centered integrated care model in China [1,2]. Within this framework, the Regional Medical Laboratory Center (RMLC) consolidates laboratory departments from 5 hospitals and 43 community health stations. Meanwhile, the central laboratory handles more complex tests with longer turnaround times, while the branch laboratories manage emergency tests and those requiring shorter turnaround times. This model seeks to optimize intraregional resource sharing, standardize testing quality, unify service standards, and promote mutual recognition of examination outcomes, thereby enhancing the overall efficiency and reliability of health care services.

Despite its many benefits, several challenges persist. Disparities in laboratory quality, differences in staff training, and the geographic spread of primary laboratories make it difficult to ensure the consistency and reliability of test results across the region [3]. Given the complexity and scale of the system, this article proposes a novel information management system designed to address these challenges. By integrating 2 key quality control (QC) strategies—internal quality control (IQC) and external quality assessment (EQA)—the system aims to enhance the consistency and accuracy of laboratory results.

IQC is an essential tool for medical laboratory quality control, continuously monitoring analytical processes to ensure their stability and reliability for patient care [4]. Medical laboratories implement defined IQC procedures, including choosing the control material, setting control limits, analyzing QC charts, detecting random errors, dealing with out-of-control reports, and managing in-house QC data [5]. Standard ISO (International Organization for Standardization) 15189:2022 (section 7.3.7.2) specifies IQC requirements, while the Clinical and Laboratory Standards Institute (CLSI) provides general guidelines for IQC implementation. The day-to-day processes of IQC are often realized through software tools, either standalone applications or those embedded within a laboratory information system (LIS) or laboratory instruments supplied [6]. However, implementing effective IQC within RMLC presents unique challenges. First, RMLC typically includes numerous laboratories with diverse equipment, procedures, and levels of staff expertise. This diversity complicates the standardization of IQC procedures across all facilities. Second, IQC data sharing among these laboratories is hindered by hospital intranet security protocols, creating information silos.

EQA is used to stimulate the harmonization and standardization processes by ensuring laboratory accuracy and monitoring the comparability of a laboratory’s testing procedures against external references over time [7]. ISO 15189:2022 (section 7.3.7.3) and CLSI QMS24 outline the requirements essential for the effective management of EQA procedures. Unfortunately, EQA programs can be expensive and time-restricted, making it challenging for basic laboratories to participate [7]. In addition to participating in EQA programs organized by government agencies, laboratories can use interlaboratory comparisons to assess their analytical capabilities and promptly address any potential issues [8-10]. Therefore, there is a need for a regional EQA system to manage the EQA process and carry out routine daily interlaboratory comparisons.

To address these challenges, we developed the iLab informatics platform, an integrated solution for IQC and EQA management within RMLC. By leveraging existing hospital network infrastructure, iLab served as a critical enabler by (1) enforcing standardized IQC and EQA protocols across the network; (2) generating objective evidence through systematic data collection and reporting to ensure compliance with ISO 15189; and (3) establishing automated, traceable workflows that met the documentation mandates of the standard. Deployed successfully in a pilot network of 5 district hospitals, 1 central laboratory, and 4 community health stations, iLab provided the technological infrastructure and standardized processes that were instrumental in achieving ISO 15189 accreditation for 206 testing items. Our findings demonstrate how strategic IT integration can transform quality management in distributed laboratory networks, offering valuable insights for health care systems aiming to standardize testing quality across multiple facilities.

## Methods

### System Requirements Analysis

The collection and analysis of QC system requirements are a critical stage that shapes the entire project’s trajectory. By thoroughly understanding the current issues, inefficiencies, and quality standards, this step defines the specific problems that need to be addressed. After conducting formal and informal interviews with laboratory quality supervisors, laboratory technicians, managers, data managers, and IT experts, the design goal of the QC system was determined. The overall goals are (1) ensuring the system functionality complies with the management requirements for IQC and EQA as defined by ISO 15189, (2) enabling seamless interconnectivity of QC data, allowing laboratories to access and use data across different institutions, and (3) achieving efficient integration of the QC system with the LIS.

### System Architecture and Components

Our IQC and EQA management approach uses 4 software programs LIS, Quality Control Box (QCBOX), Quality Control EQA Center (QCEC), and iLab electronic recording system (hereafter referred to as the iLab system). The LIS, provided by Shanghai Tengcheng Medical Tech-Info Co, Ltd, extracts QC testing data from instruments and interfaces with QCBOX for streamlined management of laboratory QC processes. The QCBOX includes functional modules such as a control dashboard, daily QC monitoring, interlaboratory comparisons, and document management. Meanwhile, the QCEC operates on a browser-server architecture, specifically designed for clinical examination centers and laboratories to efficiently manage EQA programs. The QCBOX and QCEC software, provided by Bio-Rad Laboratories (Shanghai) Co, Ltd. The custom-developed iLab electronic recording system serves as the central platform, integrating data from other components while providing specialized functions for experimental process recording, quality indicator monitoring, and continuous improvement tracking. For further details on the development principles of these software programs, please refer to Multimedia Appendix 1.

### Implementation Strategy

**Figure 1:**
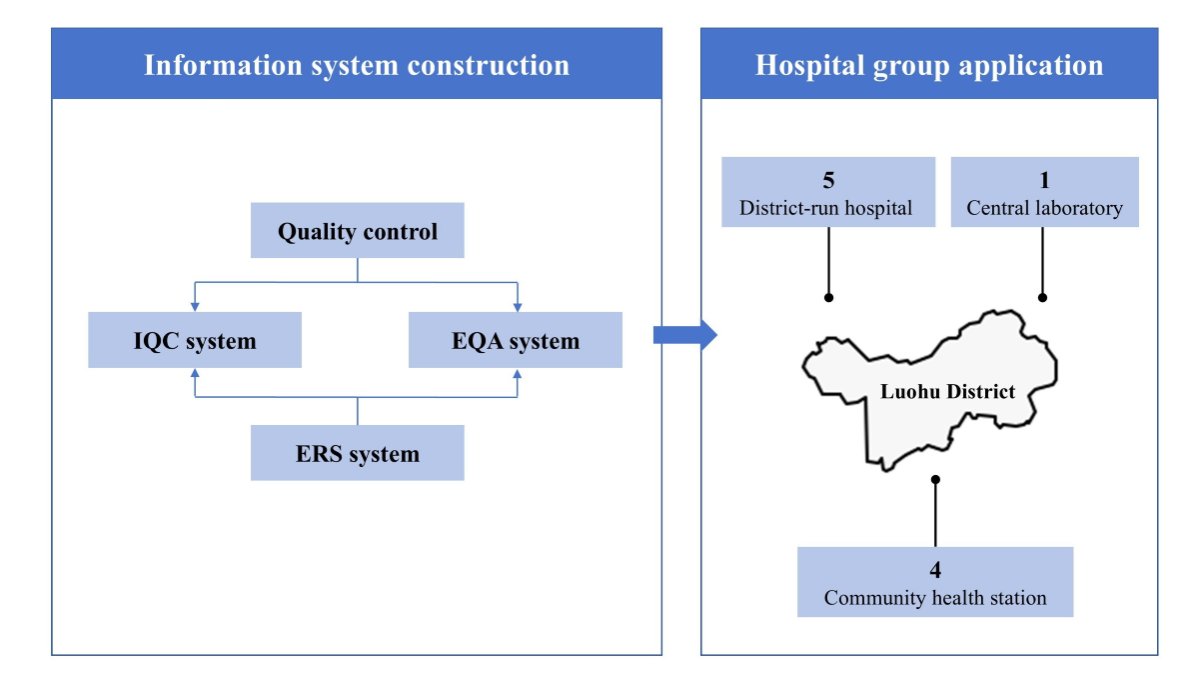
Overview of quality control process by the information system. EQA: External quality assessment; ERS: Electronic recording system; IQC: Internal quality control;.

Implementation followed a phased approach across the RMLC network, beginning with the central laboratory before extending to 5 district hospitals and 4 community health stations in Luohu District (Figure 1). The deployment process included comprehensive training sessions covering system operations, IQC failure analysis, and QC data management. Technical integration was achieved through application programming interfaces that enable real-time data exchange between the LIS, QCBOX, QCEC, and iLab systems while maintaining strict data security protocols within the hospital network infrastructure.

### Statistical Analysis

All statistical analyses were performed using R (version 4.0.3; R Core Team). We compared 4 key quality indicators between 2023 (preimplementation) and 2024 (postimplementation): the IQC implementation rate, the annual EQA item unacceptable rate, the report error rate, and the intralaboratory turnaround time compliance rate. The Pearson chi-square test was used to analyze the report error rate and turnaround time compliance rate due to the large sample sizes. Yates continuity correction was applied to the chi-square test for the IQC implementation rate. Fisher exact test was used for the annual EQA unacceptable rate to account for small cell frequencies and zero-event counts. *P* value<.05 was considered statistically significant.

### Ethical Considerations

This study was reviewed and approved by the Institutional Review Board of The Third Affiliated Hospital of Shenzhen University (Approval Number: [2025-LHQRMYY-KYLL-043]). The data were derived from routine quality management activities within the laboratory network and did not involve direct human participants. Consequently, no compensation was provided, and no personally identifiable information is included in any figures, images, or research materials presented in this study.

## Results

### Interlaboratory IQC Management Using an Information System

**Figure 2:**
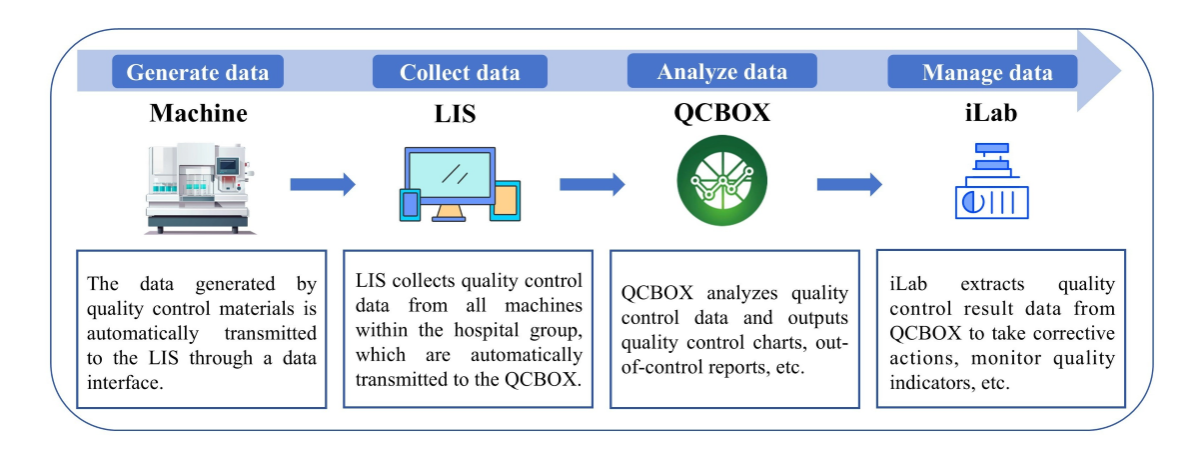
Internal quality control management by the information system. LIS: laboratory information system; QCBOX: Quality Control Box;.

IQC management encompasses 4 critical phases: data generation, data collection, data analysis, and data management. Figure 2 delineates the interconnections among these phases and the software tools instrumental in the execution. LIS serves as the central hub for collecting QC data from instruments across all laboratories and subsequently transmits this data to QCBOX. QCBOX is the software responsible for establishing QC parameters and rules, recording QC results, filing out-of-control reports, etc. The iLab system interfaces with QCBOX to streamline the automation of QC summary report reviews, enhance the monitoring of IQC-related quality indicators, and facilitate the implementation of corrective actions. This integration ensures a seamless workflow that enhances the efficiency and accuracy of the IQC process.

QCBOX automatically archives data following a comprehensive review and finalization of each IQC project. The archived information includes experimental details, QC charts, summary statements, dual review signatures, and other relevant documentation. Furthermore, technicians can access QC data for any time period or from other institutions upon demand. This empowers laboratories to comprehensively assess their overall QC performance and benchmark against external data. The interlaboratory comparison of IQC data module supports the automated retrieval of daily routine IQC data conducted in the laboratory. By comparing the IQC results with those from other regional laboratories using the same methodology and analyzing the same batch of QC materials, the module evaluates the consistency of interlaboratory results using the coefficient of variation ratio (CVR) and standard deviation index (SDI) metrics. This process is elaborated in Case Study 1. The QCBOX dashboard provides a comprehensive overview, displaying metrics such as the IQC implementation rate (percentage of IQC inspection items relative to total inspection items within the same period), the IQC coefficient of variation (CV) failure rate (percentage of inspection items with a CV higher than the requirement relative to the total items with CV requirements for IQC within the same period), and detailed information on out-of-control items for IQC within the region. Leveraging informative graphics and drill-down capabilities, this dashboard enables managers to effectively oversee IQC practices across all regional laboratories.

The iLab system is mainly used for the postanalysis management of IQC data, including four modules: (1) the QC material information record electronically records details such as the name, quantity, batch number, and expiration date; (2) the monthly summary process uses procedural form to extract monthly QC summaries from QCBOX, which undergoes a rigorous multi-stage review and approval; (3) the corrective action process uses a procedural forms to initiate a structured corrective action workflow for the out-of-control test items. The workflow meticulously documents the root cause, the corrective measures implemented, the status of corrective action completion, and subsequent verification outcomes; and (4) quality indicator monitoring, this module continuously monitors the fluctuation of 2 key metrics, IQC implementation rate and IQC CV failure rate. This monitoring is complemented by visual graphics to identify potential risks.

### Case Study 1: Interlaboratory Comparisons of Internal Quality Control Data Using QCBOX

The ISO 15189:2022 standard stipulates that interlaboratory comparisons of the results from the examination of identical IQC materials are acceptable alternatives for EQA. Regional testing centers can conduct such interlaboratory comparisons using the same IQC materials at any time through the QCBOX system. This practice holds significant value for promoting the homogenization of test results and the timely detection of potential risks. Our laboratory uses the interlaboratory comparisons feature of QCBOX for routine comparisons. As shown in Multimedia Appendix 2, we compared the consistency of potassium detection results from the central laboratory and Luohu District People’s Hospital laboratory using 4 identical instruments of the same brand and model. The laboratory’s results can be compared with the Affiliated Group (a group of laboratories that the Unity Interlaboratory Program groups together to form an ad hoc consensus group), Peer Group (all laboratories using the exact same methodology, instrument, and reagents), and Method Group (all laboratories reporting an analyte using the same methodology code). Relevant terms can be referred to in Bio-Rad’s Unity Interlaboratory Program.

Two of the most critical metrics in an interlaboratory program are the CVR and SDI, which are consensus-based metrics for imprecision and bias, respectively [11,12]. In terms of result presentation, the comparison report displays monthly and cumulative statistical data for the laboratory and the consensus groups (Affiliated, Peer, and Method Groups), encompassing parameters such as mean, SD, CV, bias, number of data points, number of laboratories, SDI, and CVR in Table S1 in Multimedia Appendix 2. The distribution of SDI or CVR can be presented through bar charts or pie charts, with automatic assessment of the laboratory testing system’s imprecision and bias based on predefined SDI or CVR thresholds [13].

### EQA Management Using Information System

**Figure 3:**
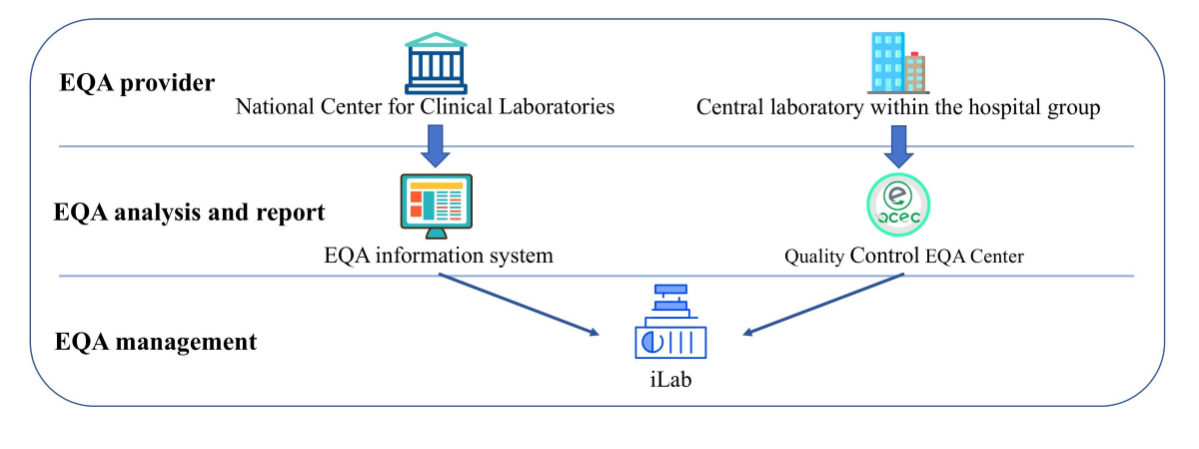
Overall workflow of the external quality assessment within the Regional Medical Laboratory Center. EQA: external quality assessment;.

EQA serves as a fundamental method for quality control and method validation in RMLC. It ensures that the results obtained from patient investigations are not only accurate and reliable but also comparable across different laboratories. Feedback from EQA results helps identify areas needing attention, such as calibration issues, procedural inconsistencies, or personnel training needs. Laboratories can then implement corrective actions to address these issues. Despite the widespread use of EQA for evaluating the quality of analytical work in medical laboratories, the role of IT in this process often receives limited attention. We therefore developed 2 IT methods to improve the EQA workflow, as depicted in Figure 3. First, we enhanced the conventional EQA processes conducted by the National Center for Clinical Laboratories (NCCL). Second, we used the QCEC software tool to operate EQA within the region, serving as a complementary approach to traditional EQA methods.

This study primarily used the iLab system to improve the conventional EQA workflow within the laboratory, focusing on elements such as electronic record-keeping, quality indicator monitoring, and detailed data analysis. Several electronic record forms were designed for shared use among regional laboratories. These forms included the EQA plan and implementation record form, interlaboratory comparison record form, interlaboratory quality assessment material receipt and processing record form, and others. Based on data from these records, we established 3 quality indicators interlaboratory, comparison rate for items without an EQA program (the proportion of items that undergo interlaboratory comparison to the total number of items without an EQA program during the same period), the EQA item coverage rate (the proportion of items participating in EQA each year relative to the total number of items with an EQA plan), and the EQA item unacceptable rate (the proportion of items participating in EQA that are deemed unacceptable each year relative to the total number of items that participated in EQA). Data trends were visualized using bar charts and line graphs. For unacceptable EQA items, we used procedural forms to document reasons for nonconformance, corrective actions taken, and verification results. For acceptable EQA items, we applied QC multirules to further analyze the data, identifying potential systematic or random errors, and proposed preventive measures accordingly [14,15]. These processes were detailed in Case Study 2.

The high cost, infrequency, and limited coverage of NCCL-administered EQA programs hindered basic laboratory participation and reduced such programs’ effectiveness as quality improvement tools [16]. To address these shortcomings, the central laboratory assumed the role of the NCCL, and QCEC serves as an effective tool for conducting EQA. Using QCEC, the central laboratory assumed the responsibilities of formulating and disseminating the EQA plan, analyzing and evaluating uploaded results and statistics, and providing feedback to division laboratories. Each laboratory was authorized to participate in the central laboratory-issued EQA plan and access the comprehensive result report. The report encompassed laboratory system information relevant to the test data, including methodology, instrumentation, reagents, and calibrators, as well as comparison details such as target values, bias, allowable ranges, evaluation outcomes, and comparative graphical representations.

### Case Study 2: Using Quality Control Multirules to Analyze Acceptable EQA Data

**Figure 4:**
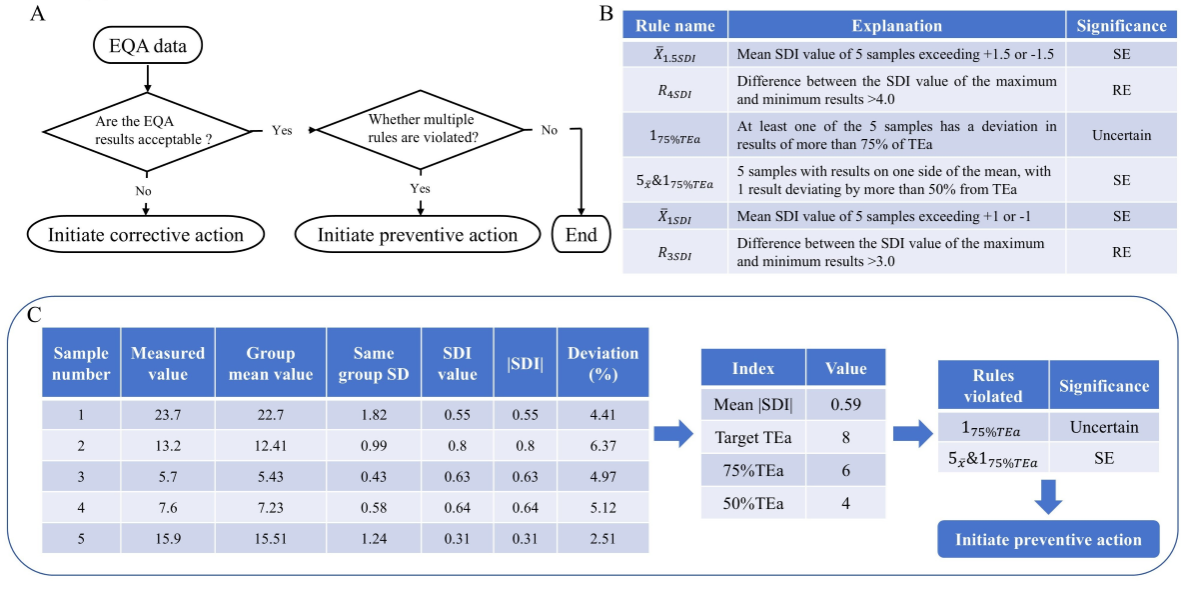
Application of the quality control multirules module to the analysis of quantitative external quality assessment data. (A) External quality assessment data review process. (B) Quality control multirules description. (C) Quality control multirules analytical process for urea satisfactory external quality assessment data. EQA: external quality assessment; RE: random error; SDI: standard deviation index; TEa: allowable total error;.

Most laboratories focus solely on analyzing unacceptable EQA data to identify root causes and implement corrective actions. However, valuable insights can be gleaned from acceptable EQA data as well. By using appropriate QC rules or rule combinations, laboratories can scrutinize acceptable EQA data to uncover potential systematic or random errors and proactively implement preventive measures (Figure 4A). Different QC rules or combinations of rules have different sensitivities to error, and the QC rules used in this laboratory and their meanings were illustrated in Figure 4B. Without dedicated software, most laboratories face significant challenges in applying QC rules due to the complexities involved in manual calculations. To address this limitation, we have introduced an EQA data multirule scheme within the iLab system.

In our laboratory, acceptable EQA results underwent additional multirule analysis incorporating 2 key metrics the SDI, derived from the difference between the measured value and group mean divided by the group SD, and the percentage deviation, calculated as the difference between measured and group mean values divided by the group mean and multiplied by 100%. Laboratory technicians simply import the measured value of the EQA, the group mean value, the same group SD, and the allowable total error (TEa) using the multirule scheme within the iLab system. The system then automatically identifies rule violations.

Figure 4C illustrates the multirule calculation process using a specific case. EQA results for urea from a particular year are presented. Upon importing the relevant data into the iLab system, it is evident that the urea results violated the 1*_75%TEa_* and rules 
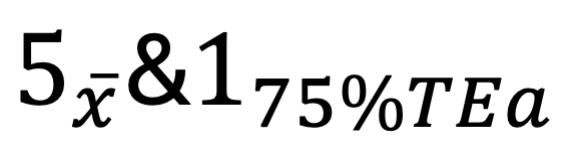
. Notably, all test results resided on the same side of the group mean value, suggesting a potential systematic error. Therefore, based on this analysis, preventive measures are necessary to calibrate the instrument and reevaluate the data following retesting of the EQA samples.

### An Integrated and Interconnected Information System for Managing IQC and EQA

**Figure 5:**
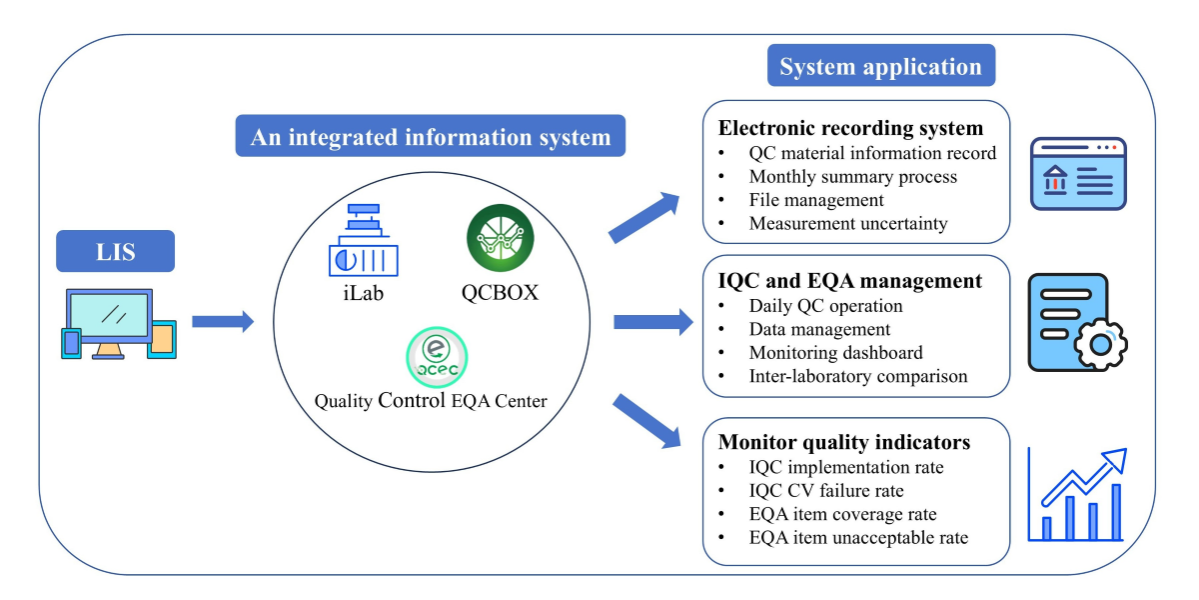
Schematic illustration of an integrated and interconnected information system for managing internal quality control and external quality assessment. CV: coefficient of variation; EQA: external quality assessment; IQC: internal quality control; LIS: laboratory information system; QC: quality control; QCBOX: Quality Control Box;.

LIS, QCBOX, QCEC, and the iLab system serve different roles within the laboratory. However, the traditional decentralized management mode often leads to issues such as information silos, resource wastage, and inefficiencies. To address these limitations, we have conducted secondary development to effectively integrate these components into a unified solution, as illustrated in Figure 5. IQC and EQA are distinct yet complementary components ensuring ongoing quality control [17]. The iLab system supports multidimensional in-depth analysis of QC data, generating valuable management information. Particularly, ISO15189:2022 mandates inclusion of measurement uncertainty (MU) calculation in all measurement procedures. We have therefore developed a tool within the iLab system based on IQC and EQA to calculate MU, detailed in Case Study 3 [18,19]. Importantly, personnel in laboratories within each division access the iLab system via authenticated accounts, facilitating interconnected data management.

### Case Study 3: Using IQC and EQA Data to Calculate MU on the Web Page

The parameter denoted by MU reflects the dispersion associated with a measured value and quantifies the reasonable level of uncertainty inherent in the result. In the context of medical laboratories, MU can be interpreted as the degree of inherent variability or inaccuracy associated with measurements obtained for a specific sample using a particular testing system or protocol. Crucially, a thorough understanding and appropriate application of MU elevate it to a key quality indicator, effectively describing the performance of both the in vitro diagnostics measuring system and the laboratory itself [20]. This empowers clinicians to use medical laboratory measurements more effectively for disease diagnosis and treatment planning.

**Figure 6:**
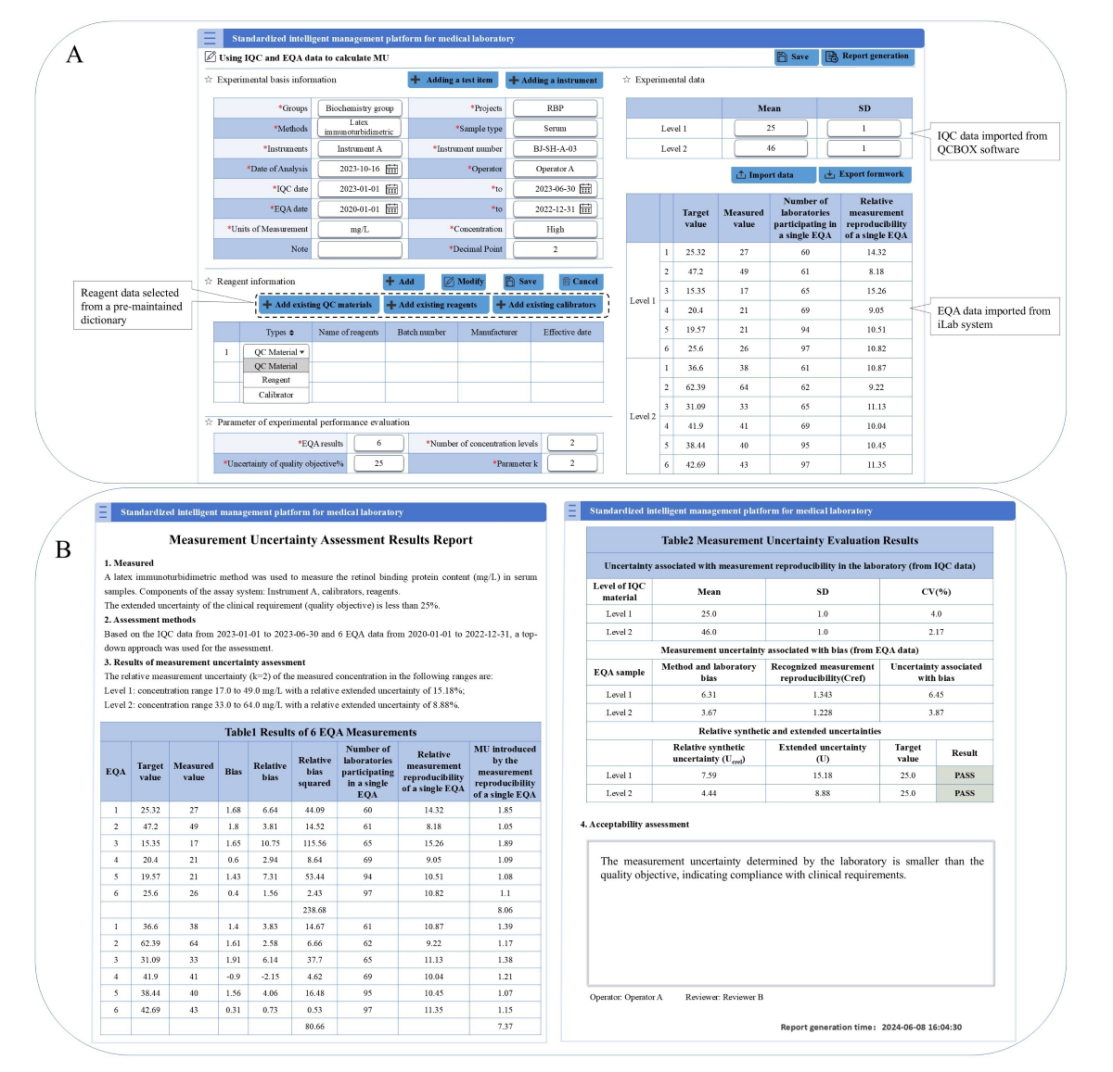
Using internal quality control and external quality assessment data to calculate measurement uncertainty on the webpage. (A) Experimental basic information entry. (B) Measurement uncertainty assessment results report. EQA: external quality assessment; IQC: internal quality control; MU: measurement uncertainty; QCBOX: Quality Control Box; RBP: retinol binding protein;.

The complexity of the MU calculation process is considered a significant barrier to its widespread adoption [21]. To efficiently evaluate the uncertainty of test items, our laboratory has developed an automatic MU evaluation function in the iLab system. As shown in Figure 6, this function includes 2 pages, the experimental information entry page (Figure 6A) and the result report page (Figure 6B).

The experimental information entry page comprises several sections:

- Basic laboratory information maintenance: this includes the entry of items to be evaluated, methodologies, instruments, and the statistical period for IQC and EQA data. It is important to note that the types of instruments and test items are maintained in a predefined dictionary, allowing for direct selection from this dictionary during entry.
- Reagent information: this involves the entry of reagent names, catalog numbers, expiration dates, etc. The types of reagents can also be selected from a premaintained dictionary.
- Parameter of experimental performance evaluation: this section includes the number of concentration levels, uncertainty of quality objective, parameter k, and other relevant parameters.
- Experimental data: this section allows for the import or manual entry of IQC and EQA data.

By inputting the appropriate data into the system, an evaluation report can be generated with one click, eliminating the need for manual calculation and significantly reducing the laboratory’s workload in MU evaluation. As shown in Figure 6B, the result report page includes a summary of experimental information, EQA statistical results, MU statistical results, and the evaluation conclusion.

Taking retinol binding protein (RBP) as an example, 2 concentrations of IQC data from 2023-01-01 to 2023-06-30 were taken to assess the MU introduced by the reproducibility of laboratory measurements (Figure 6). A total of 6 EQA results from January 1, 2020 to December 31, 2022 were used to assess the MU associated with the deviation. The mean values of the 2 concentrations of RBP for the IQC were 25 mg/L and 46 mg/L. The final results indicate that the MU at 2 concentration levels meets the quality objective.

In clinical applications, MU serves 2 primary purposes. First, it assists clinicians in decision-making when test results are close to clinical decision thresholds. For example, considering RBP with a reference range of 25-70 mg/L, when the test result is 28 mg/L, the relative expanded uncertainty at Level 1 is 15.18% (Figure 6B), yielding a 95% CI of 23.75-32.25 mg/L. In this situation, pathological factors cannot be directly excluded, and the result should be interpreted in conjunction with the patient’s symptoms and medical history. Second, MU is crucial for evaluating serial measurements, enabling clinicians to distinguish true biological trends from random analytical variation. For instance, if a patient’s consecutive test results are 71 mg/L and 68 mg/L, with a relative synthetic uncertainty of 4.44% at Level 2 (Figure 6B), the minimum difference threshold at the 95% confidence level is 8.73 mg/L. Since the observed difference of 3 mg/L is below this threshold, clinicians can infer that the variation is attributable to analytical imprecision rather than an actual change in the patient’s condition.

### Evaluation of System Implementation Effectiveness

The iLab platform achieved rapid, large-scale implementation, resulting in significant measurable gains in operational efficiency and quality performance across the RMLC network. Following its official launch in August 2023, the system integrated 133 QC instruments and 859 active QC items under 114 registered users by September 2025. This deployment was essential in establishing formalized electronic IQC, EQA, and quality management frameworks in basic-level laboratories, where such infrastructure had previously been lacking. The transition from manual to automated processes streamlined workflows and reduced staff workload. This efficiency gain is evidenced by the system’s output during this period: generating 2331 monthly IQC analysis reports, 289 MU calculation reports, and 340 qualified EQA multirule analysis reports, identifying 93 rule violations. Automation not only streamlined workflows but also ensured comprehensive digital documentation and full traceability for all quality management actions, further supported by more than 20 training sessions conducted on system operation and IQC result analysis.

Quantitative analysis of the 5 ISO15189-accredited laboratories demonstrated sustained improvements in quality performance metrics following system implementation. The IQC implementation rate significantly increased from 97.79% in 2023 to 99.87% in 2024 (*P*<.001). Operational efficiency and accuracy also showed marked gains. The report error rate declined from 0.048% to 0.027% (*P*<.001), while the intralaboratory turnaround time compliance rate improved from 95.49% to 95.71% (*P*<.001). The annual EQA item unacceptable rate decreased from 0.34% to 0%, though this reduction was not statistically significant. Additionally, the scope of accredited services expanded, with the number of testing items accredited under the ISO 15189 standard increasing from 203 in 2022 to 206 in 2024. These comprehensive results conclusively demonstrate the platform’s critical role in achieving centralized standardization, ensuring reliable results, and expanding high-quality services across the regional network.

## Discussion

### Principal Findings

This study demonstrates that the iLab informatics platform effectively addresses quality heterogeneity across the RMLC by integrating LIS, QCBOX, QCEC, and iLab into a unified management system. The platform establishes standardized workflows for IQC and EQA, enabling centralized data aggregation, automated interlaboratory comparisons, and streamlined quality governance. Key achievements include the implementation of electronic IQC and EQA systems in basic-level laboratories, enhanced EQA efficiency through multirule analysis and in-house assessment capabilities, and simplified measurement uncertainty calculation via an automated template. By ensuring full traceability while reducing administrative burden, the system provides a scalable and transferable model for standardizing quality management across distributed laboratory networks.

### Comparison to Prior Work

Despite the recognized benefits of IT in health care collaboration and resource integration, primary health care institutions often lack sufficient IT adoption [22]. The Luohu model aims to bridge this gap by fostering collaboration among health care institutions. Studies have shown that a strong informationization level is a key factor in improving the performance of the county medical community system [23]. IQC and EQA constitute the foundational pillars of QC in every clinical laboratory [4,24,25]. While various IQC implementation methods exist, including IQC modules embedded in LIS, commercial IQC software, and instrument-specific IQC software, these approaches have resulted in significant variability in practices across different laboratories [6,26-28]. For instance, Tanpaiboon et al [29], recently introduced a customized QC program that allows for updates to meet specific and evolving needs. However, their solution is limited to a biochemical genetics laboratory and does not address the complexities of RMLC. Similarly, Giannoli et al [30] used Levey-Jennings control charts with peer-group statistics to detect out-of-control errors when managing multiple instruments measuring the same analyte, a common scenario in RMLC. In contrast, we have integrated a functional template within our QCBOX software to enable interlaboratory comparisons for IQC, automatically selecting participants using the same QC materials and producing evaluation reports inclusive of the SDI and CVR values. This approach reduces both technical and operational barriers, enabling the timely detection of out-of-control instruments.

EQA schemes are a tool for providing participating laboratories with recognition and proof of meeting quality benchmarks [31,32]. However, their value is realized only when feedback drives laboratory improvement. Current research predominantly focuses on analyzing unacceptable EQA data, with limited attention to the mining and management of in-laboratory EQA datasets [33]. In this study, we addressed three key EQA challenges: (1) streamlining traditional EQA workflows via the iLab system to boost efficiency, (2) implementing multirule analysis of acceptable EQA data for automated detection of systematic or random errors, and (3) enabling in-house EQA organization within the RMLC using QCEC software, allowing flexible, autonomous proficiency testing.

While the routine reporting of MU with patient test results is not recommended by MU guidelines, it should be readily accessible upon request [34]. Recent guidelines and studies have highlighted the significance of MU estimation in medical laboratories, and the usage of IQC and EQA data for MU calculation has gained recognition as a reliable statistical method [19,34,35]. This study presents a user-friendly function template for computing MU using IQC and EQA data. This template simplifies complex calculations for technicians, enabling them to obtain results with minimal effort by simply inputting experimental data. Although the clinical applications described highlight the immense theoretical utility of MU, we acknowledge that its current primary function is to serve laboratory quality governance and compliance. Its routine adoption by frontline clinicians requires significant effort beyond mere technological support and crucially, relies on clinicians to fully understand the clinical value of MU and proactively demand this information from the laboratory.

### System Scalability and Generalizability

Our system is designed with generalizability as an important feature, and its fundamental framework is theoretically transferable to a wide range of clinical laboratories, including those in less-resourced or rural settings. However, we acknowledge that successful implementation requires three categories of basic resources that are standard in clinical laboratories and not limited to large urban networks. First, appropriate hardware infrastructure compatible with the software requirements is needed. The system operates on standard computing hardware consistent with the baseline infrastructure of most clinical laboratories, including those in resource-limited settings, without necessitating specialized high-performance computing equipment. Second, sustainable operational costs are necessary, as economic feasibility depends on covering routine expenses such as software licensing and periodic hardware maintenance. These costs are comparable to those of established laboratory workflows and can be easily incorporated into standard operational budgets. Third, personnel competence in protocol implementation is required, as effective deployment relies on laboratory staff possessing basic technical proficiency in operating the software interface and adhering to the standardized protocols described in our manuscript. This can be achieved through targeted training programs that do not demand advanced specialized expertise, ensuring adaptability across varying skill levels. In summary, although our system is inherently generalizable, its practical application presupposes access to these foundational resources.

### Limitations

This study has several limitations. First, while the system demonstrated improvements in analytical quality metrics, its downstream effects on clinical decision-making, turnaround time for critical results, or patient outcomes were not directly evaluated. Second, substantial training and workflow adaptation were required for integration, potentially hindering adoption in laboratories with limited technical support. Third, as the remaining community health stations have not yet formally adopted the system, unforeseen issues may emerge during broader deployment that were not identified in the current pilot phase.

### Conclusions

This study demonstrates that while RMLC are essential for standardizing laboratory testing, ensuring consistent result quality across diverse laboratories remains a challenge. To address this, we developed and implemented the iLab informatics platform, an integrated solution for managing IQC and EQA. The successful pilot deployment shows that our approach not only standardizes processes and supports continuous quality improvement but also provides a scalable and adaptable model, applicable to other health care networks seeking to enhance laboratory outcome consistency and integrity.

## Data Availability

The datasets generated or analyzed during this study are available from the corresponding author on reasonable request.

## Appendix

Multimedia Appendix 1 Technical architecture overview of the four software systems (LIS, QCBOX, QCEC, and iLab).

Multimedia Appendix 2 The interlaboratory comparison results using QCBOX.

## Abbreviations

CV: Coefficient of Variation
CLSI: Clinical and Laboratory Standards Institute
CVR: Coefficient of Variation Ratio
EQA: external quality assessment
LIS: laboratory information system
IQC: internal quality control
ISO: International Organization for Standardization
MU: measurement uncertainty
NCCL: National Center for Clinical Laboratories
QC: quality control
QCBOX: Quality Control Box
QCEC: Quality Control EQA Center
RBP: retinol binding protein
RMLC: Regional Medical Laboratory Center
SDI: Standard Deviation Index
TEa: allowable total error
