# Supplementary material for: Development and Implementation of an Internal Quality Control and External Quality Assessment Information System for a Regional Medical Laboratory Center: Pilot Design and Implementation Study
Source: JMIR Form Res. 2025 Dec 9;9:e77043. doi: 10.2196/77043 (PMC12728402; doi:10.2196/77043)
Supplement: Multimedia Appendix 1 [file formative_v9i1e77043_app1.docx]

**Supplemental method**

LIS is designed with a client-server-software (B/S/S) three-tier architecture at its core, aiming to achieve paperless, automated, and intelligent operations within the laboratory environment. The technical infrastructure of the LIS system includes a database server running Microsoft SQL Server 2012 or higher, a web server equipped with IIS 7.0 or above, and a .NET Framework 4.0 environment on a Windows Server 2012 or newer operating system. For the frontend, compatibility with Google Chrome version 28 and above, along with Windows 10 or later operating systems and Microsoft Office 2010 or higher, ensures smooth operation.

QCBOX uses a three-tier architecture with a Windows Presentation Foundation frontend, a business logic layer based on the Model-View-ViewModel pattern using Entity Framework for data access, and a structured SQL Server backend. The system maintains secure data management through established frameworks and supports various database components including tables, views, and stored procedures. QCEC leverages a modern web technology stack featuring a React and Tailwind CSS frontend, a .NET and C# backend with Entity Framework Core, SQL Server database management with optional Redis caching, and cross-platform deployment capabilities supporting both Windows and Linux environments.

The iLab system is implemented using a Java Web technology stack with a browser-based access interface. The backend infrastructure is developed using Java 8 with the Spring Boot framework, following a standard three-tier architecture pattern comprising Controller, Service, and Data Access Object layers. The frontend interface is constructed with Vue.js and ElementUI components, with frontend-backend communication established through HTTP APIs. For data persistence, the system employs MySQL 8 for business data storage, Redis for dictionary and cache data management, and ClickHouse for large-scale Laboratory Information System (LIS) data storage and analysis.
