# Supplementary material for: Development and Implementation of an Internal Quality Control and External Quality Assessment Information System for a Regional Medical Laboratory Center: Pilot Design and Implementation Study
Source: JMIR Form Res. 2025 Dec 9;9:e77043. doi: 10.2196/77043 (PMC12728402; doi:10.2196/77043)
Supplement: Multimedia Appendix 2 [file formative_v9i1e77043_app2.docx]

**Table S1.** The interlaboratory comparison results using QCBOX.

| **Indicator** | **Mon^a^** | **Cum^b^** | **Indicator** | **Laboratories** | | **Affiliated Group** | | **Peer Group** | | **Method Group** | |
| --- | --- | --- | --- | --- | --- | --- | --- | --- | --- | --- | --- |
|  |  |  |  | **Mon** | **Cum** | **Mon** | **Cum** | **Mon** | **Cum** | **Mon** | **Cum** |
| **Batch Number:**45950; **Items:** K | | | | | | | | | | | |
| **Laboratory:** Luohu District People's Hospital laboratory; **Instrument:** Instrument A; **Reagent:** original kit; **Unit:** mmol/L; **Methodology:** ion-selective electrode method. | | | | | | | | | | | |
| AG^c^ CVR | 0.7048 | 0.7798 | Mean | 7.6 | 7.5833 | 7.5675 | 7.5675 | 7.6181 | 7.62 | 7.5522 | 7.5559 |
| PG^d^ CVR | 0.3693 | 0.3469 | SD | 0.03 | 0.0433 | 0.045 | 0.0575 | 0.0825 | 0.09 | 0.1347 | 0.128 |
| MG^e^ CVR | 0.2242 | 0.2361 | CV | 0.4 | 0.5933 | 0.5675 | 0.7608 | 1.08 | 1.15 | 1.784 | 1.694 |
| AG SDI | 0.7222 | 0.2754 | Lab bias | Nan | Nan | 0.0043 | 0.0021 | -0.0024 | -0.0031 | 0.0063 | 0.0063 |
| PG SDI | -0.22 | -0.2723 | Points | 23 | 57 | 109 | 312 | 10008 | 118229 | 48810 | 48810 |
| MG SDI | 0.3547 | 0.3448 | Labs | Nan | Nan | 4 | 4 | 214 | 290 | 1093 | 1754 |
| **Laboratory:** Central Laboratory; **Instrument:** Instrument B; **Reagent:** original kit; **Unit:** mmol/L; **Methodology:** ion-selective electrode method. | | | | | | | | | | | |
| AG CVR | 0.8987 | 1.0515 | Mean | 7.53 | 7.5333 | 7.5675 | 7.5675 | 7.6181 | 7.6239 | 7.5522 | 7.5559 |
| PG CVR | 0.4709 | 0.4423 | SD | 0.04 | 0.06 | 0.045 | 0.0575 | 0.0825 | 0.0879 | 0.1347 | 0.128 |
| MG CVR | 0.2859 | 0.3011 | CV | 0.51 | 0.8 | 0.5675 | 0.7608 | 1.083 | 1.153 | 1.784 | 1.694 |
| AG SDI | -0.8333 | -0.5942 | Lab bias | Nan | Nan | -0.005 | -0.0045 | -0.0116 | -0.0123 | -0.0029 | -0.0029 |
| PG SDI | -1.0684 | -1.0686 | Points | 32 | 101 | 109 | 312 | 10008 | 118229 | 48810 | 48810 |
| MG SDI | -0.1648 | -0.2021 | Labs | Nan | Nan | 4 | 4 | 214 | 290 | 1093 | 1754 |
| **Laboratory:** Luohu District People's Hospital laboratory; **Instrument:** Instrument C; **Reagent:** original kit; **Unit:** mmol/L; **Methodology:** ion-selective electrode method. | | | | | | | | | | | |
| AG CVR | 1.2687 | 0.9507 | Mean | 7.6 | 7.6267 | 7.5675 | 7.5675 | 7.6181 | 7.6239 | 7.5522 | 7.5559 |
| PG CVR | 0.6648 | 0.6245 | SD | 0.06 | 0.0567 | 0.045 | 0.0575 | 0.0825 | 0.0879 | 0.1347 | 0.128 |
| MG CVR | 0.4036 | 0.425 | CV | 0.72 | 0.7233 | 0.5675 | 0.7608 | 1.083 | 1.153 | 1.784 | 1.694 |
| AG SDI | 0.7222 | 1.029 | Lab bias | Nan | Nan | 0.0043 | 0.0078 | -0.0024 | -0.0031 | 0.0063 | 0.0063 |
| PG SDI | -0.22 | -0.2723 | Points | 24 | 58 | 109 | 312 | 10008 | 118229 | 48810 | 48810 |
| MG SDI | 0.3547 | 0.3448 | Labs | Nan | Nan | 4 | 4 | 214 | 290 | 1093 | 1754 |
| **Laboratory:** Central Laboratory; **Instrument:** Instrument D; **Reagent:** original kit; **Unit:** mmol/L; **Methodology:** ion-selective electrode method. | | | | | | | | | | | |
| AG CVR | 1.1278 | 1.218 | Mean | 7.54 | 7.5267 | 7.5675 | 7.5675 | 7.6181 | 7.6239 | 7.5522 | 7.5559 |
| PG CVR | 0.591 | 0.5551 | SD | 0.05 | 0.07 | 0.045 | 0.0575 | 0.0825 | 0.0879 | 0.1347 | 0.128 |
| MG CVR | 0.3587 | 0.3778 | CV | 0.64 | 0.9267 | 0.5675 | 0.7608 | 1.083 | 1.153 | 1.784 | 1.694 |
| AG SDI | -0.6111 | -0.7101 | Lab bias | Nan | Nan | -0.0036 | -0.0054 | -0.0103 | -0.011 | -0.0016 | -0.0016 |
| PG SDI | -0.9472 | -0.9548 | Points | 30 | 96 | 109 | 312 | 10008 | 118229 | 48810 | 48810 |
| MG SDI | -0.0906 | -0.124 | Labs | Nan | Nan | 4 | 4 | 214 | 290 | 1093 | 1754 |

a: current month; b: cumulation; c: Affiliated Group; d: Peer Group; e: Method Group
